# Pain and mortality among older adults in Korea

**DOI:** 10.4178/epih.e2021058

**Published:** 2021-09-07

**Authors:** Chiil Song, Wankyo Chung

**Affiliations:** 1 Graduate School of Public Health, Seoul National University, Seoul, Korea; 2 Artificial Intelligence Institute, Seoul National University, Seoul, Korea

**Keywords:** Pain, Mortality, Proportional hazards models, Korea

## Abstract

**OBJECTIVES:**

With the increasing elderly population with chronic disease, understanding pain and designing appropriate policy interventions to it have become crucial. While pain is a noted mortality risk factor, limited studies exist due to the various causes of pain and the subjectivity of pain expression. This study aimed to examine the relationship between pain and mortality, controlling for other diseases and socio-cultural factors.

**METHODS:**

We analyzed 6,258 individuals aged 45 years or older, the population with the highest prevalence of pain, using the Korean Longitudinal Study of Aging (2006-2016) data and the Cox proportional-hazards model. Further subgroup analyses were conducted by sex and education level to examine differences in the relationship between pain and mortality.

**RESULTS:**

The adjusted hazard ratios of mortality were 1.16 (95% confidence interval [CI], 1.00 to 1.34, model 1) and 1.12 (95% CI, 0.97 to 1.29, model 2) for the individuals in pain depending on the models used, where additional socio-cultural factors were accounted for in model 2. For individuals in severe pain, ratios were significantly higher with 1.23 (95% CI, 1.08 to 1.41, model 1) and 1.16 (95% CI, 1.02 to 1.32, model 2). Further subgroup analyses showed that severe pain was more associated with mortality for males and more educated individuals, with adjusted hazard ratios of 1.29 (95% CI, 1.08 to 1.55, model 2) and 1.62 (95% CI, 1.15 to 2.28, model 2), respectively.

**CONCLUSIONS:**

Pain showed a statistically significant relationship with mortality risk. Family members or medical staff should pay proper attention to pain, particularly severe pain in males and highly educated individuals.

## INTRODUCTION

Pain, an unpleasant sensory or emotional experience associated with actual or potential tissue damage, affects not only the lives of individuals but also the society at large. The World Health Organization considers pain management to be an important factor, and they are working to develop appropriate strategies for pain management [1-4]. Due to factors such as an increase in the elderly population and the prevalence of chronic diseases, pain is affecting an increasing number of people. A review of the Korean Statistical Information Service data on pain-related diseases among the most frequent diseases (M54, R10, R51, R07, G44, G43, R52, N94, H92, R30) reveals that the number of people diagnosed with pain-related diseases in 2018 was 10.53 million, which has increased by 13.7% over the past 5 years. The medical costs of said individuals in 2018 were 1.5642 trillion Korean won, which has increased 45.0% over the same period (Supplementary Material 1). Considering the particularly steep trend of population aging in Korea, the medical expenditures caused by increasing numbers of pain-related patients will add to the financial burden of both the National Health Insurance and individual households. Therefore, there is a need to accurately understand the problems associated with pain and develop policy interventions to address them.

Pain can cause physical and psychological losses, such as disabilities and sequelae, and can also increase the risks of mortality [5-18]. In particular, patients with severe or widespread pain indicated higher mortality risks than other patients [6-11,16,17].

However, it is difficult to say whether pain directly increases the mortality risk. This is because depending on the disease that is determined to be the cause of death, the effects of pain can sometimes be insignificant, and when controlling for various external factors, the association between pain and cause of death is observed at weaker levels [13,19-21]. Furthermore, since pain is the combined output of various physical states, such as genetic, psychological states, and diseases, and the varied subjective expression by individuals and different socio-cultural factors, it is challenging to evaluate pain objectively [5,22]. Most importantly, as there are only a few prior studies on the relationship between pain and mortality and they vary widely in their methodology, there is insufficient information to clearly determine the said relationship [23].

Regardless, considering the socioeconomic effects of pain, research on the relationship between pain and mortality is crucial. This is especially true in Korea, where research is lacking in these fields. This study analyzes the effect of pain expression on the objective measure of death, and it will also consider elements, such as the diseases that affect pain and socio-cultural factors, with the intent of contributing to policies for pain and mortality management.

## MATERIALS AND METHODS

### Study population

This study utilized panel data and mortality statistics from the Korea Longitudinal Study of Ageing (KLoSA), published by the Korea Employment Information Service. KLoSA, which provides data every 2 years, started data collection in 2006. The data are sampled and collected from the middle-aged and elderly population of over 45 years of age who live in ordinary households in all regions of Korea excluding Jeju Island. This study used data from 2006 to 2016, from the first to sixth KLoSA data. From the 10,254 individuals in the panel for 2006, 6,258 individuals were selected for analysis: 1,978 dropouts and 2,018 people with missing data points were excluded.

### Measures

#### Dependent variable

The survival time of deceased individuals was determined by measuring the number of days from the starting point of data collection to the time of death. Individuals who had unknown times of death were excluded from the analysis. The survival time of survivors, on the other hand, was determined by measuring the number of days from the starting point of data collection to the time of the last panel interview conducted by the individuals.

#### Independent variable

The KLoSA surveys the existence of pain in 13 areas of the body—the head, shoulders, arms, wrists, fingers, chest, abdomen, waist, hips, legs, knees, ankles, and toes—by asking the following question, “In which of the following parts do you feel pain?”, which is then followed by sub-items that characterize the degree of pain experienced in each area. For this study, the presence of pain in one or more of the 13 areas was classified as “pain”. Likewise, the presence of severe pain in one or more of the 13 areas was classified as “severe pain”. That is, the effects of pain were analyzed based on the pain-related variables defined above: “pain” and “severe pain”.

#### Control variables

To observe the independent effects of pain on death, health variables, which are the presence of chronic disease diagnoses and disability, were controlled in the analysis. The diagnosis included the following chronic diseases: high blood pressure, diabetes, cancer, chronic pulmonary disease, liver disease, cardiovascular disease, cerebrovascular disease, psychological disease, arthritis, or rheumatism. Age and sex were also controlled for, where age was grouped into five categories: 45-54 years, 55-64 years, 65-74 years, 75-84 years, and 85 or more years.

Moreover, based on previous research, socio-cultural factors that can affect the subjective expression of pain, such as education level, income, marital status, religion, economic activity, were used as control variables [5-8,15]. Education levels were grouped into the following categories: high school or above and middle school or below. For income, the logarithm of personal gross income was used. For marital status, there were two groups: individuals not living with their spouse for various reasons, i.e., single, married but living separately, divorced, bereaved, versus individuals living with their spouse. Religion was determined from the individuals’ declaration. Economic activity was used as a control variable, which differentiated between economically active, which includes both currently employed individuals and unemployed individuals seeking employment, and economically inactive people.

### Statistical analysis

The individuals were grouped as follows: “pain” and the control group of “no pain”, and “severe pain” and the control group of “no severe pain”. Between-groups differences according to each variable were analyzed using independent t-tests and chi-square tests, with respective p-values. In addition, the difference in the survival rate between the two groups was compared using the Kaplan–Meier curve, and the statistical significance of said analysis was confirmed via log-rank tests.

The Cox proportional-hazards model was used to analyze the association between pain and mortality risk. All analyses underwent tests to confirm that they satisfied the assumptions of proportional hazards. In cases where they did not meet the assumptions of proportional hazards, they then underwent stratified analysis. In addition, all individuals were assigned into subgroups categorized by sex and educational levels, which were then individually analyzed for their relationship between pain and mortality. Stata version 13 SE (StataCorp., College Station, TX, USA) was used for statistical analysis.

### Ethics statement

Since this study uses secondary data that is publicly available, it has been categorized as “institutional review board (IRB) exempt” by the IRB of Seoul National University (IRB No. E1909/003-003).

## RESULTS

The characteristics of the individuals have been summarized in Table 1. Among all individuals, 4,099 (65.5%) responded they have pain, and 2,159 (34.5%) responded they do not. Meanwhile, 1,924 (30.7%) indicated that they have severe pain, while 4,334 (69.3%) indicated that they do not have severe pain. Among all 6,258 individuals, 5,050 (80.7%) had survived until the end of the research, while 1,208 (19.3%) individuals had died during the research period.

The mortality rate of the group who responded to have pain was 22.4%, which was higher than that of the group that indicated no presence of pain (13.4%), which was statistically significant (p<0.001). The survival time of the group who responded to have pain was 3,295.94 days, which was significantly (p<0.001) shorter than that of the group that indicated no presence of pain (3,428.95 days). Likewise, the mortality rate of the presence of severe pain group was 26.6%, which was higher than that of the control group (16.1%), which was also statistically significant (p<0.001). The survival time of the former was 3,219.38 days, which was significantly (p<0.001) shorter than that of the control group (3,396.19 days).

Both groups, specifically those with the presence of pain and severe pain, had a higher proportion of female than male, higher average age, and higher ratios of people with high blood pressure, diabetes, chronic pulmonary disease, cardiovascular disease, cerebrovascular disease, psychological disease, arthritis or rheumatism and disabilities, all with statistical significance (all p<0.001). Both groups also had relatively low education levels and income, did not live with their spouse, had a religion, and had higher ratios of economically inactive people compared to their respective control groups, which were all statistically significant (all p<0.001).

**Figure 1.**
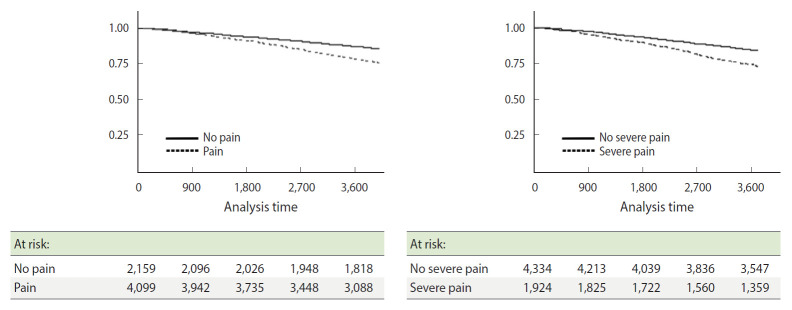
Kaplan–Meier estimate for survival rate according to pain phenotype in 2006-2016. The y-axis represents the survival rate and the x-axis represents the period of survival (in days). The numbers on the bottom are number of survivors for each group at the time of analysis.

To inspect the difference in mortality between the “pain” and “severe pain” groups, the Kaplan–Meier survival analysis was used. The survival rate of the “pain” group was observed to decrease to approximately 75% after 10 years (Figure 1). In comparison, the 10-year survival rate of the control group decreased at a slower rate; the difference between the survival rates of these two groups was observed to increase over time. The survival rate of the “severe pain” group also decreased to 75% or less after 10 years, which also showed a significant difference from the control group. The two differences between the survival rates of both groups were statistically significant (Long-rank test, all p<0.001).

To control for confounders that affect death while analyzing the relationship between pain and mortality, the Cox proportionalhazards model was used for analysis, with the results presented in Table 2. Two models were used to present the results: model 1, which controls for sex, age, chronic disease, and disability, and model 2, which adds education level, income, religion, marital status, and economic activity on top of the variables used in model 1. The results show that the hazard ratio (HR) of the “pain” group versus the control group was 1.16 (95% confidence interval [CI]: 1.00 to 1.34; p<0.05, model 1). This was also true for model 2, with added control variables, but was not statistically significant (HR, 1.12; 95% CI, 0.97 to 1.29). Compared to the control group, the HR of the “severe pain” group for model 1 was 1.23 (95% CI, 1.08 to 1.41) with statistical significance; the result was also statistically significant for model 2 with added control variables (HR, 1.16; 95% CI, 1.02 to 1.32).

Analysis of differences in the HRs according to subgroups divided by sex and education levels are presented in Table 3. For male, the HR tended to be higher for the “pain” group at 1.18 (95% CI, 0.99 to 1.40), and the “severe pain” group at 1.29 (95% CI, 1.08 to 1.55), compared to their control groups, where the result for the “severe pain” group was statistically significant. For female, the HRs were higher for the “pain” group at 1.04 (95% CI, 0.79 to 1.37) and the “severe pain” group at 1.08 (95% CI, 0.89 to 1.30), but neither of them was statistically significant.

For the “high school or above” education level group, the HRs tended to be higher for the “pain” group at 1.32 (95% CI, 0.99 to 1.75) and the “severe pain” group at 1.62 (95% CI, 1.15 to 2.28) compared to their controls, where the “severe pain” group showed statistically significant results. For the “middle school or below” education level group, compared to their controls, the HR for the “pain” group was 1.06 (95% CI, 0.89 to 1.25), and for the “severe pain” group was 1.10 (95% CI, 0.95 to 1.26), and these values were not statistically significant.

## DISCUSSION

This study used KLoSA data for observing the relationship between pain expression and mortality, since the current environment demands policy interventions for pain management, with the increasing number of patients experiencing pain. First, observing the survival rates analyzed using the Kaplan–Meier curve, the survival rates of both “pain” and “severe pain” groups were significantly lower than their controls. Moreover, using the Cox proportional-hazards model for survival analysis, “pain” group reported significantly higher HRs compared to their controls (model 1), and this coincided with the results of previous research that indicate that pain expression is associated with mortality [5-18]. In particular, mortality risk was significantly higher in the “severe pain” group (model 1: 23%; model 2: 16%). On the other hand, survival analysis for just the pain groups (“pain” and “severe pain”) indicated that the “severe pain” group had HRs that were significantly higher than the “pain” group, at 1.23 (95% CI, 1.07 to 1.42, model 1) and 1.17 (95% CI, 1.02 to 1.35, model 2), respectively (Supplementary Material 2). These results coincide with preceding research, where patients reporting the presence of severe pain showed a higher mortality risk compared to patients reporting no pain or mild pain [6,8,17]. Finally, analyzing the mortality risk between “none,” “mild pain,” and “severe pain” groups indicated that the HRs tended to increase in the aforementioned order of groups (Supplementary Material 3).

Pain is affected by various physical states, such as genetic, psychological states, and diseases, as well as other socio-cultural variables such as religion, ethnicity, culture, occupation, race, and historical background [5,22,24-28]. Therefore, it is necessary to include the various factors that affect pain in the analysis of the effects of pain on mortality [5-8]. This study observed that compared to controlling only for the factors that directly affect the health of individuals including chronic diseases, disability, sex, and age, the relationship between pain and mortality weakened when additional controlling variables of socio-cultural nature, educational level, income, religion, marital status, and economic activity are used; however, the second model still held statistical significance for the “severe pain” group.

On the other hand, even the same level or type of pain is expressed differently depending on sex and education level. Literature indicated that male are less likely to express their pain compared to female and had difficulty expressing their pain experience and severity even when they actually attempted to do so [28-30]. In particular, female expressed pain in various ways and in more areas as they got older [28]. Moreover, depending on the education level, there are differences in health behaviors, occupational environments, and access to medical care, which also affect pain expression [30]. This study analyzed subgroups of sex and found that female expressed pain more often than male (62.4 vs. 37.6%). However, it was only in the male group that the “severe pain” significantly showed increased mortality risk. According to education level subgroup analysis, “middle school or below” expressed pain more often (78.2 vs. 21.8%), but the presence of severe pain affected mortality rates with statistical significance only for the “high school or above” group.

This study analyzed the “pain” and “severe pain”; however, it has some limitations as it did not consider objective measures of pain severity or the duration of illness. Some foreign research articles use the term “widespread pain” to incorporate the number of body regions with pain to determine the severity of pain, which is then used to determine the relationship between pain and mortality [5,7-10,16]. Therefore, similar to the analysis [9], in this study, the additional analysis after defining widespread pain as pain in four or more body regions indicated that while widespread pain increased the mortality risk (model 1: HR, 1.17; 95% CI, 0.97 to 1.42; model 2: HR, 1.10, 95% CI, 0.91 to 1.33), it did not have statistical significance (Supplementary Material 4).

In addition, in order to consider the duration of illness, among 2,296 individuals without pain at the time of the first-panel survey in 2006, excluding those with pain, 1,458 individuals (63.5%) who did not continue to have pain as of the second-panel survey in 2008 and 838 individuals (36.5%) who complained of new pain were analyzed for survival analysis. We found that the “new severe pain” group had HRs of 1.77 (95% CI, 1.28 to 2.46, model 1) and 1.57 (95% CI, 1.13 to 2.18, model 2), which suggest a statistically significant increase (Supplementary Material 5). Regardless, as the items of KLoSA are not sufficiently detailed, and the study only has a limited number of individuals, these limitations need to be overcome in future research using advanced data to determine the detailed mechanisms by which pain interacts with mortality.

In the future, increases in the elderly population and chronic diseases will accelerate, and this will necessitate an accurate understanding of pain and a need for policy interventions in addressing pain. However, understanding of pain remains insufficient and pain is considered subjective, which is why medical professionals have the tendency to think that patients overexpress their degree of pain [31-33]. Therefore, this study is meaningful for having confirmed the relationship between pain and mortality in the population that most often experiences it, the middle-aged and the elderly. On the other hand, this study attempted to reflect various socio-cultural factors that can affect the subjective nature of pain expression. The results of this study indicate that family members or medical professionals must consider pain experiences to be critical factors related to mortality and that the pain expression of male and those with higher levels of education need to be considered with more concern, as they are closely related to mortality risks.

## Figures and Tables

**Table 1.** Sample characteristics at baseline in 2006

Characteristics		No pain (n=2,159)	Pain (n=4,099)	p-value	No severe pain (n=4,334)	Severe pain (n=1,924)	p-value
Death							
	No	1,869 (86.6)	3,181 (77.6)	<0.001	3,638 (83.9)	1,412 (73.4)	<0.001
	Yes	290 (13.4)	918 (22.4)		696 (16.1)	512 (26.6)	
Survival time (d)		3,428.95±710.51	3,295.94±831.21	<0.001	3,396.19±736.64	3,219.38±898.62	<0.001
Sex							
	Female	660 (30.6)	2,559 (62.4)	<0.001	1,890 (43.6)	1,329 (69.1)	<0.001
	Male	1,499 (69.4)	1,540 (37.6)		2,444 (56.4)	595 (30.9)	
Age (yr)		58.81±10.15	65.20±10.38	<0.001	61.18±10.51	67.10±10.11	<0.001
	45-54	895 (41.4)	743 (18.1)	<0.001	1,386 (32.0)	252 (13.1)	<0.001
	55-64	641 (29.7)	1,144 (27.9)		1,311 (30.2)	474 (24.6)	
	65-74	458 (21.2)	1,413 (34.5)		1,140 (26.3)	731 (38.0)	
	75-84	133 (6.2)	684 (16.7)		419 (9.7)	398 (20.7)	
	≥85	32 (1.5)	115 (2.8)		78 (1.8)	69 (3.6)	
Hypertension							
	No	1,735 (80.4)	2,726 (66.5)	<0.001	3,296 (76.0)	1,165 (60.6)	<0.001
	Yes	424 (19.6)	1,373 (33.5)		1,038 (24.0)	759 (39.4)	
Diabetes mellitus							
	No	1,973 (91.4)	3,531 (86.1)	<0.001	3,881 (89.5)	1,623 (84.4)	<0.001
	Yes	186 (8.6)	568 (13.9)		453 (10.5)	301 (15.6)	
Cancer							
	No	2,117 (98.1)	3,973 (96.9)	0.009	4,226 (97.5)	1,864 (96.9)	0.157
	Yes	42 (1.9)	126 (3.1)		108 (2.5)	60 (3.1)	
Chronic pulmonary disease							
	No	2,127 (98.5)	3,975 (97.0)	<0.001	4,253 (98.1)	1,849 (96.1)	<0.001
	Yes	32 (1.5)	124 (3.0)		81 (1.9)	75 (3.9)	
Chronic liver disease							
	No	2,129 (98.6)	4,021 (98.1)	0.138	4,272 (98.6)	1,878 (97.6)	0.007
	Yes	30 (1.4)	78 (1.9)		62 (1.4)	46 (2.4)	
Heart disease							
	No	2,098 (97.2)	3,830 (93.4)	<0.001	4,157 (95.9)	1,771 (92.0)	<0.001
	Yes	61 (2.8)	269 (6.6)		177 (4.1)	153 (8.0)	
Cerebrovascular disease							
	No	2,106 (97.5)	3,918 (95.6)	<0.001	4,210 (97.1)	1,814 (94.3)	<0.001
	Yes	53 (2.5)	181 (4.4)		124 (2.9)	110 (5.7)	
Psychological disease							
	No	2,141 (99.2)	3,288 (97.3)	<0.001	4,282 (98.8)	1,847 (96.0)	<0.001
	Yes	18 (0.8)	111 (2.7)		52 (1.2)	77 (4.0)	
Arthritis							
	No	2,019 (97.7)	3,082 (75.2)	<0.001	4013 (92.6)	1178 (61.2)	<0.001
	Yes	50 (2.3)	1017 (24.8)		321 (7.4)	746 (38.8)	
Disability							
	No	2,073 (96.0)	3,734 (91.1)	<0.001	4,125 (95.2)	1,682 (87.4)	<0.001
	Yes	86 (4.0)	365 (8.9)		209 (4.8)	242 (12.6)	
Education							
	Middle school or below	979 (45.3)	3,205 (78.2)	<0.001	2,529 (58.4)	1,655 (86.0)	<0.001
	High school or above	1,180 (54.7)	894 (21.8)		1805 (41.6)	269 (14.0)	
Per capita income (log)		6.66±1.66	5.73±1.66	<0.001	6.34±1.69	5.40±1.59	<0.001
Spouse							
	No	238 (11.0)	1,119 (27.3)	<0.001	701 (16.2)	656 (34.1)	<0.001
	Yes	1,921 (89.0)	2,980 (72.7)		3,633 (83.8)	1,268 (65.9)	
Religion							
	No	1,074 (49.7)	1,853 (45.2)	0.001	2,128 (49.1)	799 (41.5)	<0.001
	Yes	1,085 (50.3)	2,246 (54.8)		2,206 (50.9)	1,125 (58.5)	
Economic Activity							
	No	845 (39.1)	2,593 (63.3)	<0.001	2,094 (48.3)	1,344 (69.9)	<0.001
	Yes	1,314 (60.9)	1,506 (36.7)		2,240 (51.7)	580 (30.1)	

Values are presented as number (%) or mean±standard deviation.

**Table 2.** Mortality risk according to pain phenotype

Phenotype	Model 1	Model 2
Pain	1.16 (1.00, 1.34)^*^	1.12 (0.97, 1.29)
Severe pain	1.23 (1.08, 1.41)^**^	1.16 (1.02, 1.32)^*^

Values are presented as adjusted hazard ratio (95% confidence interval). Model 1: Adjusted for sex, age, chronic disease (hypertension, diabetes, cancer, chronic pulmonary disease, liver disease, heart disease, cerebrovascular disease, psychological disease, arthritis or rheumatism), and disability; Model 2: Adjusted for the variables in model 1 and also for education level, income, religion, marital status, and economic activity.

* p<0.05,

** p<0.01.

**Table 3.** Mortality risk according to pain phenotype by sex and educational level^1^

Variables	Phenotype	
	Pain	Severe pain
Sex		
Female (n=3,219)	1.04 (0.79, 1.37)	1.08 (0.89, 1.30)
Male (n=3,039)	1.18 (0.99, 1.40)	1.29 (1.08, 1.55)^**^
Education		
Middle school of below (n=4,184)	1.06 (0.89, 1.25)	1.10 (0.95, 1.26)
High school or above (n=2,074)	1.32 (0.99, 1.75)	1.62 (1.15, 2.28)^**^

Values are presented as adjusted hazard ratio (95% confidence interval).

1 Adjusted for sex, age, chronic diseases (hypertension, diabetes, cancer, chronic pulmonary disease, liver disease, heart disease, cerebrovascular disease, psychological disease, arthritis or rheumatism), disability, education level, income, religion, marital status, and economic activity.

** p<0.01.

## SUPPLEMENTARY MATERIALS

Korean version is available at https://www.e-epih.org/.
