# Supplementary material for: Pain and mortality among older adults in Korea
Source: Epidemiol Health. 2021 Sep 7;43:e2021058. doi: 10.4178/epih.e2021058 (PMC8666684; doi:10.4178/epih.e2021058)
Supplement: Supplementary file 1 [file epih-43-e2021058-suppl1.docx]

**Supplementary Materials**

| **Supplementary Material 1. Changes in the number of patients and medical expenses for**  **pain-related diseases** | | |
| --- | --- | --- |
| Year | No. of patients treated (person) | Medical expenses (thousand won) |
| 2014 | 9,258,120 | 1,078,453,425 |
| 2015 | 9,439,960 | 1,150,863,140 |
| 2016 | 9,967,587 | 1,278,979,776 |
| 2017 | 10,150,564 | 1,394,125,963 |
| 2018 | 10,529,822 | 1,564,203,010 |

Codes for pain-related diseases are M54, R10, R51, R07, G44, G43, R52, N94, H92, and R30. https://kosis.kr/statHtml/statHtml.do?orgId=350&tblId=DT_35001_A074111&conn_path=I2.

| **Supplementary Material 2. Mortality risk using the mild pain group as a reference group** | | | |
| --- | --- | --- | --- |
|  | Unadjusted HR  (95% CI) | Adjusted HR  (95% CI, Model 1) | Adjusted HR  (95% CI, Model 2) |
| Mild pain | Ref. | Ref. | Ref. |
| Severe pain | **1.50(1.32-1.71)***** | **1.23 (1.07-1.42)**** | **1.17 (1.02-1.35)*** |

The Cox proportional-hazards model was used on 4,099 subjects excluding the non-pain group. The mild pain group were 2,175 individuals (53.1%), and the severe pain group were 1,924 individuals (46.9%).

Model 1: Adjusted for gender, age, chronic disease (hypertension, diabetes, cancer, chronic pulmonary disease, liver disease, heart disease, cerebrovascular disease, psychological disease, arthritis or rheumatism), and disability

Model 2: Adjusted for the variables in Model 1 and also for education level, income, religion, marital status, and economic activity

*p < 0.05, **p < 0.01, ***p < 0.001

| **Supplementary Material 3. Mortality risk across three pain groups** | | | |
| --- | --- | --- | --- |
|  | None | Mild pain | Severe pain |
| None | - | 1.06 (0.91-1.24) | **1.21 (1.02-1.43)*** |

Adjusted for gender, age, chronic disease (hypertension, diabetes, cancer, chronic pulmonary disease, liver disease, heart disease, cerebrovascular disease, psychological disease, arthritis or rheumatism), disability, education level, income, religion, marital status, and economic activity (Model 2)

*p < 0.05, **p < 0.01, ***p < 0.001

| **Supplementary Material 4. Mortality risk by number of pain sites** | | | |
| --- | --- | --- | --- |
| No. of sites | Unadjusted HR  (95% CI) | Adjusted HR  (95% CI, Model 1) | Adjusted HR  (95% CI, Model 2) |
| None | Ref. | Ref. | Ref. |
| 1-3 (Regional pain) | **1.64 (1.43-1.88)***** | 1.15 (0.99-1.33) | 1.12 (0.97-1.30) |
| 4≥ (Widespread pain) | **2.10 (1.78-2.49)***** | 1.17 (0.97-1.42) | 1.10 (0.91-1.33) |

Model 1: Adjusted for gender, age, chronic disease (hypertension, diabetes, cancer, chronic pulmonary disease, liver disease, heart disease, cerebrovascular disease, psychological disease, arthritis or rheumatism), and disability

Model 2: Adjusted for the variables in Model 1 and also for education level, income, religion, marital status, and economic activity

*p < 0.05, **p < 0.01, ***p < 0.001

| **Supplementary Material 5. Mortality risk according to new pain phenotype (base year=2008)** | | | |
| --- | --- | --- | --- |
|  | Unadjusted HR  (95% CI) | Adjusted HR  (95% CI, Model 1) | Adjusted HR  (95% CI, Model 2) |
| New Pain | **1.64 (1.29-2.11)***** | 1.21 (0.93-1.57) | 1.13 (0.87-1.47) |
| Severe new pain | **3.10 (2.27-4.22)***** | **1.77 (1.28-2.46)***** | **1.57 (1.13-2.18)**** |

The Cox proportional-hazards model was used on 2,296 individuals without pain in Wave 1 (2006). In 2008 (Wave 2) 1,458 (63.5%) were still without pain but 838 (36.5%) felt new pain.

Model 1: Adjusted for gender, age, chronic disease, and disability

Model 2: Adjusted for the variables in Model 1 and also for education level, income, religion, marital status, and economic activity

*p < 0.05, **p < 0.01, ***p < 0.001
