# Supplementary material for: Pain and mortality among older adults in Korea
Source: Epidemiol Health. 2021 Sep 7;43:e2021058. doi: 10.4178/epih.e2021058 (PMC8666684; doi:10.4178/epih.e2021058)
Supplement: Supplementary file 2 [file epih-43-e2021058-suppl2.docx]

**Title**: Pain and mortality among older adults in Korea

**Full names of all authors**: Chiil Song, Wankyo Chung

**Affiliations:** Graduate School of Public Health, Seoul National University

**ORICD**: (Chiil Song) 0000-0002-1150-1885

(Wankyo Chung) 0000-0001-8094-2433

**Corresponding author's contact information**

Wankyo Chung,

Professor

Graduate School of Public Health & SNU AI Institute (AIIS)

Seoul National University

(08826) 1, Gwanak-ro, Gwanak-gu, Seoul, Republic of Korea

Graduate School of Public Health

E-mail: wankyo@snu.ac.kr

Tel: +82-2-880-2285

**Running title**: Pain and mortality among older adults in Korea

**Pain and mortality among older adults in Korea**

**Abstract**

*Objectives*

With the increasing elderly population with chronic disease, understanding pain and designing appropriate policy interventions to it have become crucial. While pain is a noted mortality risk factor, limited studies exist due to the various causes of pain and the subjectivity of pain expression. This study aimed to examine the relationship between pain and mortality, controlling for other diseases and sociocultural factors.

*Methods*

We analyzed 6,258 individuals aged 45 years or older, the population with the highest prevalence of pain, using the Korean Longitudinal Study of Aging (2006–2016) data and the Cox proportional-hazards model. Further subgroup analyses were conducted by sex and education level to examine differences in the relationship between pain and mortality.

*Results*

The adjusted hazard ratios of mortality were 1.16 (95% CI: 1.00–1.34, Model 1) and 1.12 (95% CI: 0.97–1.29, Model 2) for the individuals in pain depending on the models used, where additional sociocultural factors were accounted for in Model 2. For individuals in severe pain, ratios were significantly higher with 1.23 (95% CI: 1.08–1.41, Model 1) and 1.16 (95% CI: 1.02–1.32, Model 2). Further subgroup analyses showed that severe pain was more associated with mortality for males and more educated individuals, with adjusted hazard ratios of 1.29 (95% CI: 1.08–1.55, Model 2) and 1.62 (95% CI: 1.15–2.28, Model 2), respectively.

*Conclusions*

Pain showed a statistically significant relationship with mortality risk. Family members or medical staff should pay proper attention to pain, particularly severe pain in males and highly educated individuals.

**key words**

Pain, Mortality, Proportional Hazards Models, Korea

1. Introduction

실재 혹은 잠재적 조직손상으로 인한 기분 나쁜 감각이나 감정적 경험인 통증은 개인의 삶뿐만 아니라 사회에도 큰 영향을 끼치고 있어, 세계 보건기구에서는 통증 문제를 중요하게 인식하고 적절한 통증 관리를 위해 노력하고 있다[1-4]. 하지만 통증은 인구고령화와 만성질환의 증가로 점점 더 많은 사람에게 영향을 미치고 있다. KOSIS의 다빈도 상병 자료 중 통증 상병(M54, R10, R51, R07, G44, G43, R52, N94, H92, R30)과 관련된 자료를 보면, 우리나라에서 통증으로 인한 질환의 진료실인원은 2018년의 1,053만 명으로 최근 5년 동안 13.7% 증가하였으며, 이들의 진료비는 2018년의 1조 5642억원으로 같은 기간 동안 45.0% 증가하였다(부표 1). 특히 우리나라의 인구고령화 추세를 고려한다면, 통증 인구의 증가로 인한 가계 및 건강보험 재정 부담은 더욱 가중될 것이다. 따라서 통증 문제에 대해 정확히 이해하고 그에 따른 정책적 접근이 요구되는 상황이다.

통증은 장애 및 후유증과 같은 신체적 그리고 정신적인 손실을 초래할 수 있으며, 사망의 위험을 증가시키기도 한다[5-18]. 특히 심각한 통증 혹은 많은 신체부위의 통증을 호소하는 환자들은 그 외 환자들보다 사망위험이 높은 것으로 나타났다[6-11, 16, 17].

하지만 통증이 사망을 직접적으로 높인다고 단정하기는 어렵다. 사망의 원인이 되는 질환에 따라 통증의 영향이 유의하지 않거나, 다양한 외부 요인을 통제하는 경우 통증과 사망 간의 연관성이 약하게 관찰되기 때문이다[13, 19-21]. 또한 통증은 유전, 정신적 상태, 질환과 같은 다양한 신체 상태의 복합적 결과물이자 개인의 주관적인 표현으로, 개인의 사회문화적인 상황에 따라 표현의 차이가 존재하기 때문에 객관적으로 평가하기 어렵다[5, 22, 23]. 무엇보다 통증과 사망 간의 관계에 대한 선행연구가 적고, 이전에 실행된 연구들의 방법론적인 차이 때문에 그 관계를 명확하게 판단하기에는 부족함이 있다[24].

하지만 통증의 사회경제적 영향을 고려할 때 통증과 사망 간의 관계에 대한 연구는 중요하며, 특히 관련 연구가 부족한 우리나라에서는 더욱 중요하다. 본 연구는 통증 표현이 객관적 지표인 사망에 미치는 영향을 분석하며, 나아가 통증에 영향을 미치는 질환과 사회문화적인 요소를 고려하여 성별과 학력에 따른 하위그룹별 분석을 함으로써 통증과 사망 관리 정책에 기여하고자 한다.

2. Methods

1) Study population

본 연구는 한국고용정보원에서 발간하는 고령화연구패널조사(Korea Longitudinal Study of Ageing)의 패널 자료와 사망자 자료를 분석에 사용하였다. 고령화연구패널조사는 2006년에 조사를 시작하였으며, 제주도를 제외한 지역에 거주하는 45세 이상 중·고령자 중 일반가구 거주자를 대상으로 표집 및 조사를 실시하여 매 2년 간격으로 데이터를 제공하고 있다. 본 연구는 2006년부터 2016년까지의 6차년도의 데이터를 사용하였다. 2006년의 패널 대상자 10,254명중 중도 탈락자 1,978명과 사망 시기를 알 수 없거나 데이터에 결측값이 있는 2,018명을 제외한 6,258명을 분석대상자로 선정하였다.

2) Ethics Statement

본 연구는 공개된 2차 자료를 이용하는 연구로 서울대학교 생명윤리위원회로부터 심의 면제 대상으로 승인 받았다(IRB No. E1909/003-003).

3) Measures

3.1) Dependent variable

사망자의 생존기간은 분석자료의 시작시점부터 사망이 일어난 시점까지의 생존기간을 일단위로 측정하였으며, 사망한 시기를 알 수 없는 경우 분석대상에서 제외하였다. 한편 생존자의 생존기간은 분석자료의 시작시점부터 마지막 패널 조사의 응답시점까지의 생존일수로 계산하였다.

3.2) Independent variable

고령화연구패널조사는 “다음 중 어느 부위에 통증을 느끼십니까?” 라는 질문으로 머리, 어깨, 팔, 손목, 손가락, 가슴, 배, 허리, 엉덩이, 다리, 무릎, 발목, 발가락 등 총 13개 부위의 통증 여부를 물으며, 각 부위의 통증 정도를 묻는 하위 문항을 제공한다. 본 연구에서는 13개 부위 중 한 개 부위 이상에 통증을 호소하면 통증이 있는 것으로 정의하였다. 마찬가지로 13개 부위 중 한 개 부위 이상에 심한 통증을 표현하면 심한 통증이 있는 것으로 정의하였다. 즉 통증 여부, 심한 통증 여부에 따라 통증 변수를 정의하여 통증의 영향을 살펴보았다.

3.3) Control variables

통증이 사망에 미치는 독립적인 영향을 살펴보기 위해 만성질환진단과 장애 여부를 건강 변수로 통제하였다. 본 연구에서는 만성질환 변수로 고혈압, 당뇨, 암, 만성폐질환, 간질환, 심장질환, 뇌혈관질환, 정신과적질환, 관절염 또는 류머티즘질환의 진단 여부를 사용하였다. 성별과 나이를 통제하였으며, 나이는 45-54세, 55-64세, 65-74세, 75-84세, 85세 이상의 구간으로 나누어 분석에 사용하였다.

또한 선행연구를 바탕으로 통증의 주관적인 표현에 영향을 미칠 수 있는 사회문화적인 요인인 학력과 소득 및 배우자 유무, 종교 유무, 경제활동참여 여부 등을 통제변수로 사용하였다[5-8, 15]. 학력은 고등학교 졸업 및 그 이상 그룹과 중학교 졸업 이하 그룹으로 나누었으며, 소득은 Log를 취한 개인 총소득 값을 이용하였다. 배우자 유무는 배우자가 현재 있는 그룹과 별거, 이혼, 사별 및 미혼으로 현재 배우자가 없는 그룹으로 구분하였고, 종교 유무는 종교 여부 응답에 따라 구분하였다. 경제활동 여부는 취업자와 구직자를 포함하는 경제활동인구와 비경제활동인구로 구분하여 통제변수로 사용하였다.

4) Statistical analysis

먼저 연구대상자를 통증 집단과 그 대조군, 심한 통증집단과 그 대조군으로 나누어 각 변수에 따른 두 집단간의 차이를 독립 t 검정 및 chi-square 검정을 시행하고 p값을 제시하였다. 또한 두 집단간 생존율의 차이를 Kaplan-Meier 곡선을 이용하여 비교하고 Log-rank test를 이용하여 통계적 유의성을 확인하였다.

통증이 사망위험을 높이는지 연관성을 분석하기 위해서는 Cox 비례위험모델(Cox Proportional-Hazards Model)을 사용하였다. 모든 분석은 비례 위험의 가정을 만족하는지 적합도 검정을 시행하였으며 비례 위험의 가정을 만족시키지 못하는 경우 층화 분석을 시행하였다. 더불어 전체 연구대상자를 성별과 학력 수준에 따라 하위그룹으로 나누어, 하위그룹별로 통증과 사망 간의 관계를 분석하였다. 분석에는 STATA version 13 SE (StataCorp)를 사용하였다.

3. Results

본 연구에 사용된 대상자의 특성은 표 1에 정리되어 있다. 전체 대상자 중 통증이 있다고 대답한 사람은 4,099명(65.5%)이며 통증이 없다고 대답한 사람은 2,159명(34.5%)이었다. 심한 통증이 있다고 응답한 사람은 1,924명(30.7%) 이었으며 심한 통증이 없다고 대답한 사람은 4,334명(69.3%)이었다. 전체 대상자 6,258명 중 연구종료시점까지 생존한 사람은 5,050명으로 80.7%였으며, 연구 기간 내 사망자는 1,208명으로 19.3%이었다.

통증이 있다고 응답한 집단의 사망률은 22.4%로 통증이 없는 집단의 사망률인 13.4%에 비하여 통계적으로 유의하게 높게 나타났다(p<0.001). 또한 통증이 있다고 응답한 집단의 생존기간은 3,295.9일로 통증이 없는 집단의 생존기간인 3,429.0일에 비해 통계적으로 유의하게 짧게 나타났다(p<0.001). 마찬가지로 심한 통증이 있는 집단의 사망률은 26.6%로 대조군에(16.1%)에 비해 유의하게 높게 나타났으며, 생존기간 역시 3219.4일로 대조군의 3396.2일에 비해 유의하게 짧게 나타났다(all p<0.001).

통증 및 심한 통증이 있는 집단에는 여성의 비율이 높고 평균연령이 높았으며, 고혈압, 당뇨, 만성폐질환, 심장질환, 뇌혈관질환, 정신과적질환, 관절염 또는 류마티스질환, 장애를 가진 사람들의 비율이 유의하게 높게 나타났다(all p<0.001). 또한 통증 및 심한 통증이 있는 집단의 교육수준은 상대적으로 낮고 소득은 적었으며, 배우자가 없고 종교가 있으며 비경제활동인구인 비율이 유의하게 높게 나타났다(all p<0.001).

통증과 심한 통증에 따른 생존율의 차이를 확인하기 위하여 Kaplan–Meier 생존곡선으로 비교하였다. 그 결과 시간이 지남에 따라 통증이 있다고 응답한 집단의 생존율은 10년 후 75% 정도까지 낮아지는 것을 확인할 수 있었다(그림 1). 그에 비해 통증이 없는 집단의 10년 후 생존율은 상대적으로 완만하게 낮아지는 것으로 나타나, 시간이 지남에 따라 두 집단 간 생존율의 차이가 커지는 것을 확인할 수 있었다. 또한 심한 통증 집단의 생존율도 10년 후 75%이하로 줄어드는 것을 확인할 수 있었으며, 대조군과 확연한 차이를 보였다. 두 집단의 생존율 차이는 통계적으로 유의한 결과를 보였다(Long-rank test, all p<0.001).

사망에 영향을 미치는 관련요인을 통제하고 통증과 사망과의 관계를 분석하기 위하여 cox 비례위험모델을 이용하여 분석하였으며, 그 결과는 표 2에 제시되어 있다. 성별, 나이, 만성질환, 장애 여부를 통제한 Model 1과 추가적으로 학력, 소득, 종교 유무, 배우자 유무, 경제활동여부를 통제한 Model 2를 사용하여 분석결과를 제시하였다. 분석결과 통증이 없는 집단에 비하여 통증이 있다고 응답한 집단에서의 사망위험비는 1.16(Model 1, 95% CI: 1.00-1.34)으로 나타났다(p<0.05). 이러한 경향은 통제변수를 추가한 분석(Model 2)에서도 나타났으나, 통계적으로 유의하지는 않았다(HR 1.12, 95% CI: 0.97-1.29). 한편 심한 통증이 있다고 응답한 집단은 그렇지 않은 집단에 비하여 사망위험비가 1.23으로 나타났으며(Model 1, 95% CI: 1.08-1.41), 이는 추가적인 변수를 통제한 경우에서도 통계적으로 유의한 결과를 보였다(Model 2, HR 1.16, 95% CI: 1.02-1.32).

한편, 통증에 따른 사망위험비가 성별과 교육수준에 따른 하위 그룹별로 어떻게 달라지는지 Model 2를 이용하여 분석한 결과는 표 3, 4에 제시되었다. 남성의 경우 통증 및 심한 통증이 있다고 응답한 집단의 사망위험비가 1.18(95% CI: 0.99-1.40)과 1.29(95% CI: 1.08-1.55)로 대조군에 비하여 높아지는 경향을 보였으며, 심한 통증의 경우 통계적으로 유의하게 높은 결과를 보였다. 여성의 경우 각각의 분석에서 사망위험비가 1.04(95% CI: 0.79-1.37)와 1.08(95% CI: 0.89-1.30)로 나타났으며 그 결과값은 통계적으로 유의하지 않았다.

또한, 고등학교 졸업 이상의 학력을 가진 집단의 경우에는 통증과 심한 통증에 따른 사망위험비가 1.32(95% CI: 0.99-1.75)와 1.62(95% CI: 1.15-2.28)로 대조군에 비하여 높아지는 경향을 보였으며, 심한 통증의 경우 통계적으로 유의하게 높은 결과를 보였다. 중학교 졸업 이하의 학력을 가진 집단에서는 통증과 심한 통증에 따른 사망위험비가 1.06(95% CI: 0.89-1.25)와 1.10(95% CI: 0.95-1.26)으로 나타났으며, 그 결과값들은 통계적으로 유의하지 않았다.

4. Discussion

본 연구는 통증 인구의 증가로 통증에 대한 정책적 접근이 요구되는 상황에서 통증 표현과 사망 간의 관계를 확인하고자 고령화연구패널 자료를 이용하여 분석을 실시하였다. 먼저 Kaplan-Meier 곡선을 통해 통증에 따른 생존율을 살펴본 결과, 통증 집단 및 심한 통증 집단의 생존율이 대조군에 비해 유의하게 낮아지는 것을 확인할 수 있었다. 또한 Cox 비례위험모델을 이용해 생존 분석을 실시한 결과, 통증을 표현한 집단의 사망위험비가 대조군에 비해 유의하게 높아지는 것을 확인할 수 있었으며(Model 1), 이러한 결과는 통증 표현이 사망과 관련이 있다는 선행연구의 결과와 일치한다[5-18]. 특히 심한 통증집단에서 사망위험이 뚜렷하게 높아지는 결과를 확인할 수 있었다[23%(Model 1), 16%(Model 2)]. 한편, 통증과 심한 통증만을 따로 구분하여 생존분석을 시행한 결과, 통증(경증)에 비해서도 심한 통증의 사망위험비가 1.23(Model 1, 95% CI: 1.07-1.42)과 1.17(Model 2, 95% CI: 1.02-1.35)로 유의하게 높은 것을 확인할 수 있었다(부표 2). 이는 선행연구에서 통증이 없거나 경증의 통증을 호소하는 집단보다 심한 통증을 호소하는 경우 사망위험이 높아지는 결과와 일치한다[6, 8, 17]. 마지막으로 비통증집단, 경증 통증집단, 심한 통증집단 간의 사망위험을 분석한 경우 비통증집단 대비 경증 및 심한 통증집단의 사망위험비는 단계적으로 증가하는 경향을 보였다(부표 3).

통증은 유전, 정신적 상태, 질환과 같은 다양한 신체 상태의 영향을 받으며, 종교, 민족, 문화, 직업군, 인종, 시대적 상황과 같은 여러 가지 사회문화적인 요인에 영향을 받는다[5, 22, 25-29]. 따라서 통증에 영향을 미치는 다양한 요인을 사망과의 분석 모델에 포함할 필요가 있다[5-8]. 본 연구에서는 건강상태에 직접적으로 영향을 미치는 만성질환, 장애, 성별, 나이 만을 통제한 경우에 비하여, 교육, 소득, 종교, 배우자 유무, 경제활동유무 등 사회문화적인 요소를 추가적으로 통제한 경우, 통증과 사망 간의 관계가 약해지나, 심한 통증의 경우에는 여전히 통계적으로 유의한 결과를 보였다.

한편 같은 통증일지라도 성별과 교육수준에 따라 통증 표현 양상이 다르게 나타난다. 선행 연구에 따르면 여성에 비해 남성은 통증을 표현할 가능성이 적으며, 통증 표현을 하는 경우 통증 경험과 통증 정도를 설명하는데 어려움을 호소했다[29-32]. 특히 나이가 들수록 여성이 호소하는 통증 부위가 남성보다 더 다양하고 많았다[29]. 또한 교육수준에 따라 건강 행동, 직업 환경, 의료 접근성 등에 차이가 발생하고, 그로 인해 통증 표현에도 차이가 있었다[32]. 본 연구에서 성별에 따른 하위 그룹 분석을 시행한 결과를 살펴보면 남성집단보다 여성집단이 통증을 더 많이 표현하나(62.4% 대 37.6%), 남성집단에서만 심한 통증이 사망위험을 통계적으로 유의하게 높이는 것으로 나타났다. 학력 수준에 따른 하위 그룹 분석의 경우 중학교 졸업 이하의 학력집단에서 통증을 더 많이 호소하였으나(78.2% 대 21.8%), 고등학교 졸업 이상의 집단에서만 심한 통증이 사망에 유의한 영향을 미치는 것으로 나타났다.

한편 본 연구가 통증과 심한 통증을 분석하였지만 객관적인 척도에 따른 통증의 정도 및 유병기간을 고려하지 못한 한계가 있다. 일부 해외 연구는 widespread pain이라는 개념을 통해 통증 부위의 개수에 따라 통증의 정도를 판단하고 사망과의 연관성이 있는지 분석하고 있다[5, 7-10, 16]. 따라서 Andersson HI[9]의 연구와 같이 widespread pain을 4부위 이상의 통증으로 정의하여 추가적 분석을 시행한 결과, widespread pain이 사망위험을 높이지만(Model 1 HR 1.17 95% CI: 0.97-1.42, Model2 HR 1.10 95% CI: 0.91-1.33), 통계적으로 유의한 결과를 보이지는 않았다(부표 4).

또한 통증의 유병기간을 고려하기 위해 2006년 첫 번째 패널조사 당시 통증이 있는 사람을 제외하고 통증이 없는 사람 2,296명 가운데, 2008년 두 번째 패널조사 시점을 기준으로 통증이 계속 없는 사람 1458명(63.5%)과 새로 통증을 호소한 사람 838명(36.5%)을 이용하여 생존분석을 시행하였다. 그 결과 새로운 심한 통증집단에서 사망위험비가 1.77(Model1, 95% CI: 1.28-2.46)과 1.57(Model2, 95% CI: 1.13-2.18)로 통계적으로 유의하게 높아지는 것을 확인할 수 있었다(부표 5). 그럼에도 불구하고 고령화연구패널조사의 통증문항이 구체적이지 못하고 샘플이 작은 한계가 있어, 향후 이를 보완한 자료를 이용하여 통증이 사망과 어떠한 기제를 통하여 서로 관계되는지에 대한 추가 연구가 필요하다.

미래에는 고령인구와 만성질환의 증가가 가속화됨에 따라 통증을 제대로 이해하고 통증 인구에 대한 적절한 정책적 접근이 요구되는 상황이다. 하지만 아직까지 통증에 대한 이해가 부족하고, 통증 자체는 객관적이지 못하다는 인식이 있으며, 특히 의료진은 환자의 통증 표현이 과대 표현되었다고 생각하는 경우가 많다[33-35]. 따라서 통증을 주로 겪는 중·고령층을 대상으로 통증이 사망에 미치는 영향을 직접적으로 확인하였다는 점에서 본 연구의 의의가 있다. 한편 본 연구에서는 통증의 주관적인 특성에 영향을 미칠 수 있는 다양한 사회문화적 요인을 분석에 반영하고자 노력하였다. 본 연구의 결과, 환자의 통증 표현을 접하는 가족 혹은 의료진들은 통증을 사망과 관련이 있는 중요한 지표로 받아들여야 하며, 특히 남성과 고학력자 집단이 표현하는 통증은 사망의 위험성과 관련이 크므로 중요하게 관찰 및 관리해야 할 필요가 있다.

5. Reference

1. Woolf AD, Erwin J, March, L. The need to address the burden of musculoskeletal conditions. Best practice & research Clinical rheumatology 2012;26:183-224.

2. Lee YG. Definition and Classification of Pain. Clinical Pain 2002;1:1-4

3. WHO. Guidelines for the pharmacological and radiotherapeutic management of cancer pain in adults and adolescents;2019

4. WHO. Guideline for the management of chronic pain in children;2020

5. McBeth J, Symmons D, Silman A, Allison T, Webb R et al. Musculoskeletal pain is associated with a long-term increased risk of cancer and cardiovascular-related mortality. Rheumatology 2008;48:74-77

6. Torrance N, Elliott AM, Lee AJ, Smith BH. Severe chronic pain is associated with increased 10year mortality. A cohort record linkage study. European journal of pain 2010;14:380-386.

7. Macfarlane GJ, Barnish MS, Jones GT. Persons with chronic widespread pain experience excess mortality: longitudinal results from UK Biobank and meta-analysis. Annals of the rheumatic diseases 2017;76:1815-1822

8. Smith D, Wilkie R, Croft P, McBeth J. Pain and mortality in older adults: the influence of pain phenotype. Arthritis care & research 2018;70:236-243.

9. Andersson HI. Increased mortality among individuals with chronic widespread pain relates to lifestyle factors: a prospective population-based study. Disability and rehabilitation 2009;31:1980-1987.

10. Tesarz J, Eich W, Baumeister D, Kohlmann T, D'Agostino R et al. Widespread pain is a risk factor for cardiovascular mortality: results from the Framingham Heart Study. European heart journal 2019;40:1609-1617

11. Zhu K, Devine A, Dick IM, Prince RL. Association of back pain frequency with mortality, coronary heart events, mobility, and quality of life in elderly women. Spine 2007;32:2012-2018

12. Kåreholt I, Brattberg G. Pain and mortality risk among elderly persons in Sweden. Pain 1998;77:271-278.

13. Smith, BH, ElliottbAM, Hannaford PC. Pain and subsequent mortality and cancer among women in the Royal College of General Practitioners Oral Contraception Study. Br J Gen Pract 2003;53:45-46

14. Nitter AK, Forseth KØ. Mortality rate and causes of death in women with self-reported musculoskeletal pain: Results from a 17-year follow-up study. Scandinavian journal of pain 2013;4:86-92.

15. Roseen EJ, LaValley MP, Li S, Saper RB, Felson DT et al. Association of back pain with all-cause and cause-specific mortality among older women: a cohort study. Journal of general internal medicine 2019;34:90-97.

16. Macfarlane GJ, Crombie IK, McBeth J, Silman AJ. Widespread body pain and mortality: prospective population based study Commentary: An interesting finding, but what does it mean?. Bmj 2001;323:662.

17. Roseen EJ, Rajendran I, Stein P, Fredman L, Fink HA et al. Association of Back Pain with Mortality: a Systematic Review and Meta-analysis of Cohort Studies. Journal of General Internal Medicine 2021;1-11

18. Tsai MH, Tsay WI, Her SH, Ho CH, Chen YC et al. Long-term mortality in older adults with chronic pain: a nationwide population-based study in Taiwan. European Geriatric Medicine 2019;10:777-784

19. Wolfe F, Hassett AL, Walitt B, Michaud K. Mortality in fibromyalgia: A study of 8,186 patients over thirty‐five years. Arthritis care & research 2011;63:94-101.

20. Breivik H, Collett B, Ventafridda V, Cohen R, Gallacher D. Survey of chronic pain in Europe: prevalence, impact on daily life, and treatment. European journal of pain 2006;10:287-287.

21. Andrews JS, Cenzer IS, Yelin E, Covinsky KE. Pain as a risk factor for disability or death. Journal of the American Geriatrics Society 2013;61:583-589.

22. Marc S. La douleur - Un exposé pour comprendre, un essai pour réfléchir. [Dominos](https://www.decitre.fr/collection/dominos); 1994

23. Park MH, Baek SH, Kim NC, Song HH. Expression of Cancer Pain in Terminal Cancer Patients. Korean Society for Hospice and Palliative Care: Journal of Hospice and Palliative Care 2003;98-98.

24. Smith D, Wilkie R, Uthman O, Jordan JL, McBeth J. Chronic pain and mortality: a systematic review. PloS one 2014;9:e99048.

25. Edwards CL, Fillingim RB, Keefe F. Race, ethnicity and pain. Pain 2001;94:133-137.

26. Chapman WP, Jones CM. Variations in cutaneous and visceral pain sensitivity in normal subjects. The Journal of clinical investigation 1944:23:81-91.

27. Martinelli AM. Pain and ethnicity. AORN journal, 1987;46:273-274.

28. Schiefenhövel W. Perception, expression, and social function of pain: a human ethological view. Science in Context 1995;8:31-46.

29. Andersson HI, Ejlertsson G, Leden I, Rosenberg C. Chronic pain in a geographically defined general population: studies of differences in age, gender, social class, and pain localization. The Clinical journal of pain 1993;9:174-182.

30. Brooks-Brunn JA, Kesler KA. Gender differences in self-reported pain following thoracic resection. Chest 2000;118;205s-205s.

31. Miller C, Newton SE. Pain perception and expression: the influence of gender, personal self-efficacy, and lifespan socialization. Pain Management Nursing 2006;7:148-152.

32. Dionne CE, Von Korff M, Koepsell TD, Deyo RA, Barlow WE et al. Formal education and back pain: a review. Journal of Epidemiology & Community Health 2001;55:455-468.

33. Kim HJ, Park IS, Kang KJ. Knowledge and awareness of nurses and doctors regarding cancer pain management in a tertiary hospital. Asian Oncology Nursing 2012;12:147-155

34. Byun JS, Choi JY. Pain Intensity, Pain Control and Pain Control Barriers between Cancer Patients and their Nurses. Asian Oncology Nursing 2013;13.

35. Carr E. Barriers to effective pain management. Journal of Perioperative Practice 2007;17:200-208.

**Figure legends**

**Figure 1** Kaplan-meier estimate for survival rate according to pain phenotype (Year 2006-2016)

The y-axis represents the survival rate and the x-axis represents the period of survival (in days). The numbers on the bottom are number of survivors for each group at the time of analysis.

**Tables**

| **Table 1 Sample characteristics at baseline (Year=2006), n (%)** | | | | | | |
| --- | --- | --- | --- | --- | --- | --- |
| **Characteristics** | **No pain**  **(n=2159)** | **Pain**  **(n=4099)** | **P-value** | **Not severe pain (n=4334)** | **Severe pain (n=1924)** | **P-value** |
| **Death** |  |  |  |  |  |  |
| No | 1869 (86.57) | 3181 (77.60) | <0.001 | 3638 (83.94) | 1412 (73.39) | <0.001 |
| Yes | 290 (13.43) | 918 (22.40) |  | 696 (16.06) | 512 (26.61) |  |
| **Survival time** |  |  |  |  |  |  |
| Mean (day) | 3428.95$\pm$710.5 | 3295.94$\pm$831.2 | <0.001 | 3396.19$\pm$736.6 | 3219.38$\pm$898.6 | <0.001 |
| **Gender** |  |  |  |  |  |  |
| Female | 660 (30.57) | 2559 (62.43) | <0.001 | 1890 (43.61) | 1329 (69.07) | <0.001 |
| Male | 1499 (69.43) | 1540 (37.57) |  | 2444 (56.39) | 595 (30.93) |  |
| **Age** |  |  |  |  |  |  |
| Mean (year) | 58.81$\pm$10.2 | 65.20$\pm$10.4 | <0.001 | 61.18$\pm$10.5 | 67.10$\pm$10.1 | <0.001 |
| 45-54 | 895 (41.46) | 743 (18.13) | <0.001 | 1386 (31.98) | 252 (13.10) | <0.001 |
| 55-64 | 641 (29.69) | 1144 (27.91) |  | 1311 (30.25) | 474 (24.64) |  |
| 65-74 | 458 (21.21) | 1413 (34.47) |  | 1140 (26.30) | 731 (37.99) |  |
| 75-84 | 133 (6.16) | 684 (16.69) |  | 419 (9.67) | 398 (20.68) |  |
| Above 85 | 32 (1.48) | 115 (2.80) |  | 78 (1.80) | 69 (3.59) |  |
| **Hypertension** |  |  |  |  |  |  |
| No | 1735 (80.36) | 2726 (66.50) | <0.001 | 3296 (76.05) | 1165 (60.55) | <0.001 |
| Yes | 424 (19.64) | 1373 (33.50) |  | 1038 (23.95) | 759 (39.45) |  |
| **Diabetes mellitus** |  |  |  |  |  |  |
| No | 1973 (91.38) | 3531 (86.14) | <0.001 | 3881 (89.55) | 1623 (84.36) | <0.001 |
| Yes | 186 (8.62) | 568 (13.86) |  | 453 (10.45) | 301 (15.64) |  |
| **Cancer** |  |  |  |  |  |  |
| No | 2117 (98.05) | 3973 (96.93) | 0.009 | 4226 (97.51) | 1864 (96.88) | 0.157 |
| Yes | 42 (1.95) | 126 (3.07) |  | 108 (2.49) | 60 (3.12) |  |
| **Chronic pulmonary disease** |  |  |  |  |  |  |
| No | 2127 (98.52) | 3975 (96.97) | <0.001 | 4253 (98.13) | 1849 (96.10) | <0.001 |
| Yes | 32 (1.48) | 124 (3.03) |  | 81 (1.87) | 75 (3.90) |  |
| **Chronic liver disease** |  |  |  |  |  |  |
| No | 2129 (98.61) | 4021 (98.10) | 0.138 | 4272 (98.57) | 1878 (97.61) | 0.007 |
| Yes | 30 (1.39) | 78 (1.90) |  | 62 (1.43) | 46 (2.39) |  |
| **Heart disease** |  |  |  |  |  |  |
| No | 2098 (97.17) | 3830 (93.44) | <0.001 | 4157 (95.92) | 1771 (92.05) | <0.001 |
| Yes | 61 (2.83) | 269 (6.56) |  | 177 (4.08) | 153 (7.95) |  |
| **Cerebrovascular disease** |  |  |  |  |  |  |
| No | 2106 (97.55) | 3918 (95.58) | <0.001 | 4210 (97.14) | 1814 (94.28) | <0.001 |
| Yes | 53 (2.45) | 181 (4.42) |  | 124 (2.86) | 110 (5.72) |  |
| **Psychological disease** |  |  |  |  |  |  |
| No | 2141 (99.17) | 3288 (97.29) | <0.001 | 4282 (98.80) | 1847 (96.00) | <0.001 |
| Yes | 18 (0.83) | 111 (2.71) |  | 52 (1.20) | 77 (4.00) |  |
| **Arthritis** |  |  |  |  |  |  |
| No | 2019 (97.68) | 3082 (75.19) | <0.001 | 4013 (92.59) | 1178 (61.23) | <0.001 |
| Yes | 50 (2.32) | 1017 (24.81) |  | 321 (7.41) | 746 (38.77) |  |
| **Disability** |  |  |  |  |  |  |
| No | 2073 (96.02) | 3734 (91.10) | <0.001 | 4125 (95.18) | 1682 (87.42) | <0.001 |
| Yes | 86 (3.98) | 365 (8.90) |  | 209 (4.82) | 242 (12.58) |  |
| **Education** |  |  |  |  |  |  |
| Middle school or below | 979 (45.35) | 3205 (78.19) | <0.001 | 2529 (58.35) | 1655 (86.02) | <0.001 |
| High school or above | 1180 (54.65) | 894 (21.81) |  | 1805 (41.65) | 269 (13.98) |  |
| **Per capita income** |  |  |  |  |  |  |
| Mean (log) | 6.66$\pm$1.7 | 5.73$\pm$1.7 | <0.001 | 6.34$\pm$1.7 | 5.40$\pm$1.6 | <0.001 |
| **Spouse** |  |  |  |  |  |  |
| No | 238 (11.02) | 1119 (27.30) | <0.001 | 701 (16.17) | 656 (34.10) | <0.001 |
| Yes | 1921 (88.98) | 2980 (72.70) |  | 3633 (83.83) | 1268 (65.90) |  |
| **Religion** |  |  |  |  |  |  |
| No | 1074 (49.75) | 1853 (45.21) | 0.001 | 2128 (49.10) | 799 (41.53) | <0.001 |
| Yes | 1085 (50.25) | 2246 (54.79) |  | 2206 (50.90) | 1125 (58.47) |  |
| **Economic Activity** |  |  |  |  |  |  |
| No | 845 (39.14) | 2593 (63.26) | <0.001 | 2094 (48.32) | 1344 (69.85) | <0.001 |
| Yes | 1314 (60.86) | 1506 (36.74) |  | 2240 (51.68) | 580 (30.15) |  |

| **Table 2 Mortality risk according to pain phenotype** | | |
| --- | --- | --- |
|  | Adjusted HR (95% CI, Model 1) | Adjusted HR (95% CI, Model 2) |
| Pain | **1.16 (1.00-1.34)*** | 1.12 (0.97-1.29) |
| Severe pain | **1.23 (1.08-1.41)**** | **1.16 (1.02-1.32)*** |

Model 1: Adjusted for gender, age, chronic disease (hypertension, diabetes, cancer, chronic pulmonary disease, liver disease, heart disease, cerebrovascular disease, psychological disease, arthritis or rheumatism), and disability

Model 2: Adjusted for the variables in Model 1 and also for education level, income, religion, marital status, and economic activity

*p < 0.05, **p < 0.01, ***p < 0.001

| **Table 3 Mortality risk according to pain phenotype by gender** | | |
| --- | --- | --- |
|  | Female (n=3,219) | Male (n=3,039) |
| Pain | 1.04 (0.79-1.37) | 1.18 (0.99-1.40) |
| Severe pain | 1.08 (0.89-1.30) | **1.29 (1.08-1.55)**** |

Adjusted for gender, age, chronic diseases (hypertension, diabetes, cancer, chronic pulmonary disease, liver disease, heart disease, cerebrovascular disease, psychological disease, arthritis or rheumatism), disability, education level, income, religion, marital status, and economic activity

*p < 0.05, **p < 0.01, ***p < 0.001

| **Table 4 Mortality risk according to pain phenotype by education level** | | |
| --- | --- | --- |
|  | Middle school or below (n=4,184) | High school or above (n=2,074) |
| Pain | 1.06 (0.89-1.25) | 1.32 (0.99-1.75) |
| Severe pain | 1.10 (0.95-1.26) | **1.62 (1.15-2.28)**** |

Adjusted for gender, age, chronic diseases (hypertension, diabetes, cancer, chronic pulmonary disease, liver disease, heart disease, cerebrovascular disease, psychological disease, arthritis or rheumatism), disability, education level, income, religion, marital status, and economic activity

*p < 0.05, **p < 0.01, ***p < 0.001
